# Atypical Endobronchial Carcinoid with Postobstructive Pneumonia Obscuring the Diagnosis of Granulomatosis with Polyangiitis

**DOI:** 10.1155/2015/513602

**Published:** 2015-08-11

**Authors:** Robert Ali, Candice Baldeo, Jesse Onyenekwe, Roshan Lala, Cristian Landa, Anwer Siddiqi

**Affiliations:** ^1^Department of Internal Medicine, University of Florida, Jacksonville, USA; ^2^Department of Pathology, University of Florida, Jacksonville, USA

## Abstract

Granulomatosis with polyangiitis (GPA), previously termed Wegener's Granulomatosis, is an autoimmune small vessel vasculitis which is highly associated with antineutrophil cytoplasmic antibodies (ANCA) and has varied clinical manifestations. Diagnosis hinges on identifying a combination of clinical features of systemic vasculitis, positive ANCA serology, and histological evidence of necrotizing vasculitis, necrotizing glomerulonephritis, or granulomatous inflammation from a relevant organ biopsy. The American College of Rheumatology has also developed a classification criteria focusing specifically on nasal or oral inflammation, abnormal chest radiograph, and abnormal urinary sediment, along with granulomatous inflammation, which helps to distinguish GPA from other forms of systemic vasculitis. In the case presented below, the diagnosis of GPA was delayed as the patient had a concomitant atypical endobronchial carcinoid which predisposed to postobstructive pneumonia. Fortunately, the papular lesions that developed across her lower limbs prompted further investigations. The return of appropriate serology coincided with progression to alveolar hemorrhage, offering a more complete clinical picture, and when she responded to the combination of steroid, cyclophosphamide, and plasma exchange, the diagnosis of GPA was cinched.

## 1. Introduction

Granulomatosis with polyangiitis (GPA), previously termed Wegener's Granulomatosis, is an autoimmune small vessel vasculitis which is highly associated with antineutrophil cytoplasmic antibodies (ANCA) and has varied clinical manifestations, including systemic necrotizing vasculitis, necrotizing granulomatous inflammation, and necrotizing glomerulonephritis [1]. The average incidence of GPA is 5–10 cases per million populations per year, with a balanced distribution between male and female populations [2]. The reported peak incidence of GPA is the 7th decade of life, with a greater prevalence among Caucasians as opposed to the Asians, Afro-Caribbeans, and African Americans [2–4]. Multiple triggers have been postulated for the development of GPA in genetically susceptible individuals, with noted increased susceptibility to proteinase-3 ANCA associated vasculitis (AAV) with certain genetic variants [2, 4]. The inaugural lesions are thought to commence as a localized airway granulomatosis that subsequently transforms to a vasculitis [5]. Effective management of GPA centers on both induction and maintenance phases of therapy. Current regimens involve the combination of glucocorticoids with immunosuppressants, such as cyclophosphamide, methotrexate, or azathioprine, with biologic agents such as rituximab now finding a foothold in both acute and chronic management of this condition [6–9].

## 2. Case

A 44-year-old Caucasian female with a history of asthma, rhinitis, and generalized joint pains presented for evaluation of recurrent dyspnea. She was initially admitted two months prior for suspected pneumonia. CT angiogram (CTA) showed diffuse bilateral pulmonary infiltrates and a large left mainstem endobronchial mass with a small focal area of lung parenchymal consolidation distal to the lesion. Bronchoscopy confirmed the presence of a partially obstructing mass and biopsy was positive for carcinoid tumor. She was treated with ceftriaxone and levofloxacin and was subsequently discharged with planned follow-up at the surgical outpatient clinic for evaluation of tumor resection. The patient subsequently lost insurance, precluding this assessment.

**Figure 1:**
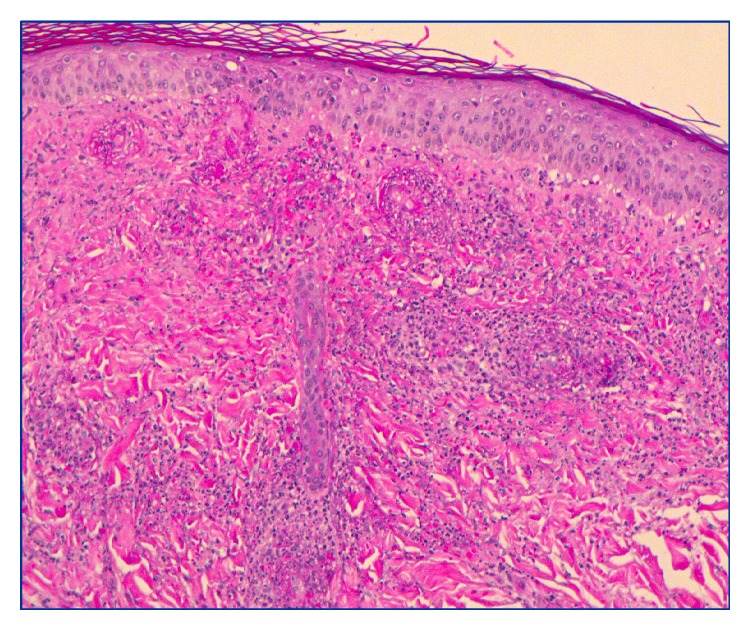
Skin biopsy showing classic features of small vessel vasculitis with neutrophilic infiltration of the vessel walls, karyorrhexis, and fibrinoid necrosis of vessel walls as well as extensive extravasation of red blood cells.

She presented six weeks later with worsening dyspnea but also noted a palpable petechial rash across both lower limbs. Chest X-ray (CXR) showed diffuse bilateral pulmonary opacities concerning multifocal pneumonia. CTA was negative for embolus but showed bilateral ground glass opacities. Skin biopsy revealed leukocytoclastic vasculitis (see Figure 1). Initial serology was positive for c-ANCA (1 : 320), but unremarkable for ANA, rheumatoid factor, p-ANCA, IgE, C3/C4, hepatitis, HIV, Lyme disease, and antistreptolysin O. CRP and ESR were mildly elevated. She was treated with levofloxacin for suspected obstructive pneumonia and was discharged after five days.

**Figure 2:**
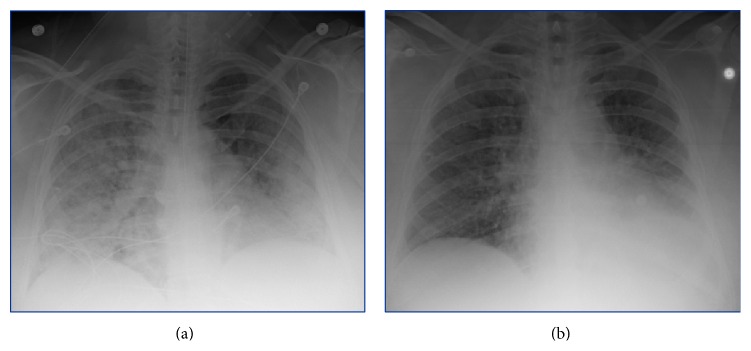
(a) CXR from admission showing bilateral extensive pulmonary infiltrates, suggestive of florid vasculitis. (b) CXR upon discharge emphasizing resolution of the pulmonary infiltrates following treatment with plasmapheresis, induction cyclophosphamide, and high dose methylprednisone.

**Figure 3:**
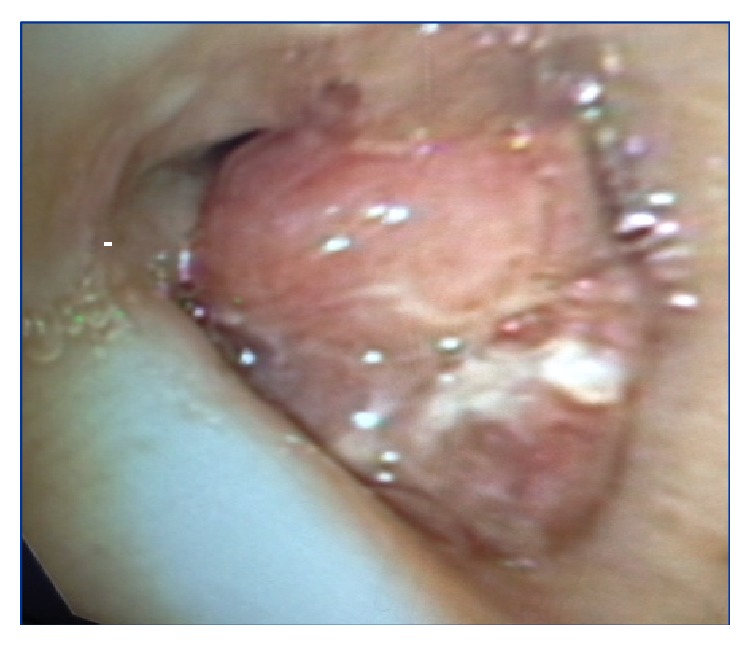
Bronchoscopy image showing the gross carcinoid tumor in the left mainstem bronchus.

**Figure 4:**
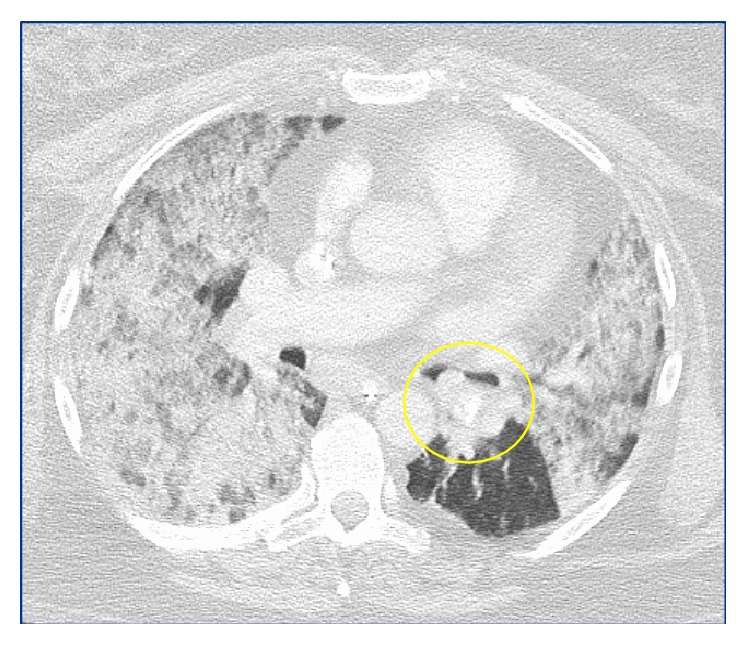
CTA chest showing bilateral ground glass opacities and highlighting the left endobronchial lesion (yellow circle).

**Figure 5:**
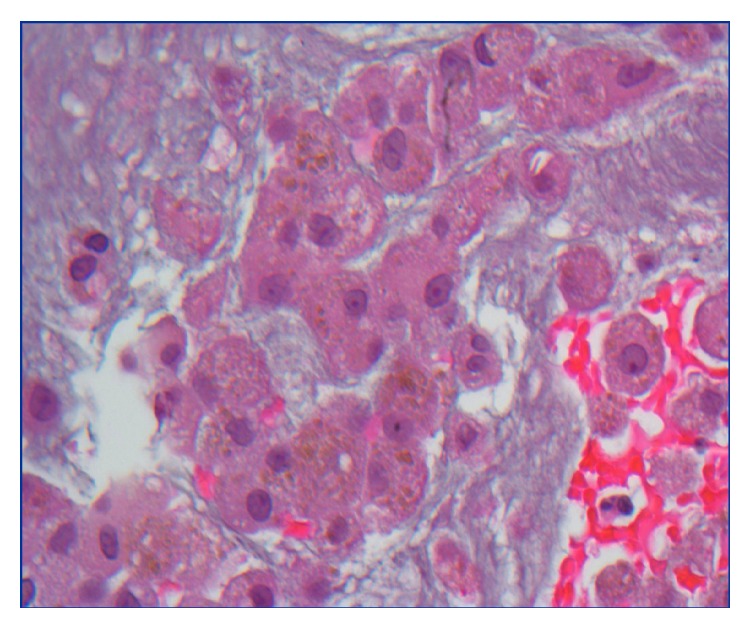
Cell block section of bronchoalveolar lavage showing hemosiderin-laden macrophages (granular golden pigment) demonstrating alveolar hemorrhage.

She presented again two weeks later with worsening dyspnea, fever, and a nonproductive cough. She was hypoxic to 85% on room air that was responsive to supplemental oxygen and had bilateral, diffuse rhonchi on examination. She had a leukocytosis of 15,000/mm^3^ and CXR showed stable bilateral pulmonary infiltrates (see Figure 2(a)). Her creatinine was elevated from a normal baseline at 1.38 mg/dL and the urinalysis was positive for 54 red blood cells/HPF. She was empirically treated for health-care associate pneumonia with Vancomycin, Zosyn, and Tobramycin. A 24-hour trial of octreotide was administered as there was concern for a carcinoid exacerbation; however she continued to deteriorate. Follow-up CXR showed worsening pulmonary infiltrates and her gas exchange worsened despite aggressive diuresis and intravenous methylprednisolone. She was subsequently intubated for hypoxic respiratory failure. Repeat bronchoscopy was positive for diffuse alveolar hemorrhage and about 90% obstruction of the left mainstem bronchus secondary to the endobronchial carcinoid (see Figures 3 and 5). Pan-culture was negative and all antibiotics were discontinued. Urinalysis was positive for 54 red blood cells/HPF, with mild elevation in creatinine. CTA showed worsening pulmonary ground glass and patchy opacities throughout both lungs (see Figure 4). Serology was negative for antiglomerular basement membrane and myeloperoxidase antibodies but positive for PR3 antibody. She was uptitrated to high dose methylprednisolone 1 gram daily for 3 days, received one dose of induction cyclophosphamide 1500 mg, and underwent one session of plasmapheresis. She had a dramatic improvement with corresponding near resolution on her chest film (see Figure 2(b)). She was subsequently extubated and eventually discharged on prednisone 60 mg daily with a prolonged taper and oral cyclophosphamide 200 mg daily. She also received concomitant* Pneumocystis jiroveci* pneumonia prophylaxis with Bactrim.

Repeat chest CT scan nearly four months later revealed a significant improvement/resolution of the diffuse opacities throughout the bilateral lungs but commented on the stable 3 cm endobronchial mass of the left lower lobe bronchus, with slightly worsened distal consolidation of the left lower lobe.

**Figure 6:**
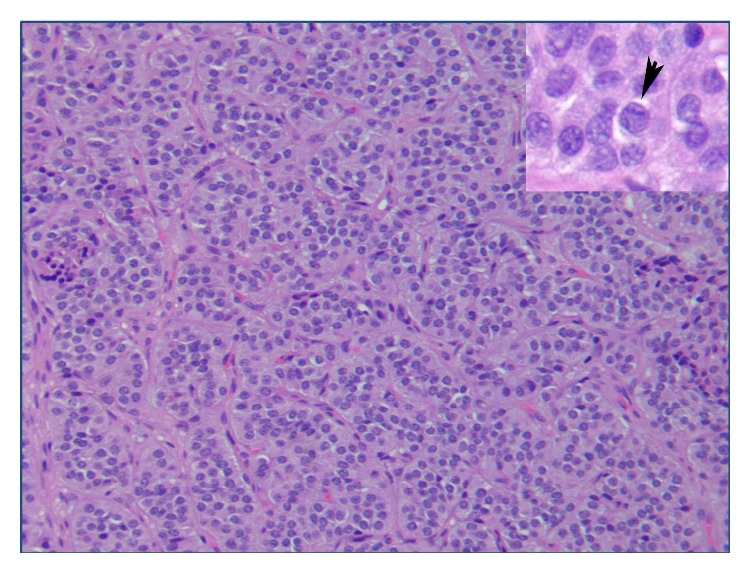
Histology slide from the atypical carcinoid tumor. Nests of uniform, bland carcinoid cells with central nuclei and moderate cytoplasm, prominent vasculature surrounding the nests (H&E, ×20). Inset: infrequent mitoses (H&E, ×63).

She subsequently underwent snare cautery, followed by argon photocoagulation debulking of the tumor. After procedure, there was more than 95% patency of the left mainstem bronchus. Origins for the left upper lobe and lingua could be easily identified; however the origin of the left lower lobe was likely encased by tumor. Pathology revealed an atypical carcinoid with 8 mitoses per 50 HPF (see Figure 6).

The cardiothoracic service evaluated the patient thereafter stating that the tumor appeared to have extended into the left upper bronchus and that sleeve resection would not be an option. Furthermore, given her poor performance status and risk of impaired wound healing while on steroids she was a poor surgical candidate.

Follow-up PET and octreotide scans were equivocal for metastatic mediastinal adenopathy but confirmed no other disease spread. She subsequently received three cycles of cisplatin/etoposide with concurrent radiotherapy. Shortly after her third cycle she developed another flare of the GPA and was restarted on high dose prednisone 60 mg daily and cyclophosphamide. The exacerbation acutely progressed, resulting in respiratory failure secondary to diffuse alveolar hemorrhage. Unfortunately, the patient succumbed the same day.

Autopsy revealed the atypical carcinoid tumor involving the left mainstem bronchus, bronchopneumonia, interstitial lung disease, and extensive intra-alveolar hemorrhage. There was noted cardiomegaly with four-chamber dilatation but no evidence of acute myocardial infarction. The liver was enlarged with steatosis, and both the lungs and spleen demonstrated congestion. In summary, her death was believed to be as a result of respiratory failure secondary to extensive pulmonary hemorrhage in a background of interstitial lung disease and a clinical history of granulomatosis with polyangiitis.

## 3. Discussion

Diagnosis of GPA is not always straightforward, especially when confounded by other disease processes. Furthermore, clinical manifestations of GPA can be highly varied and are dependent upon which organ system is affected by the vasculitis (see Table 1). Our case models this stipulation, as the presence of a nearly completely obstructive endobronchial lesion muddled the respiratory features, and the ongoing atypical carcinoid tumor evoked the rare but possible occurrence of a carcinoid syndrome. That being said, upper airway involvement is nearly ubiquitous, and pulmonary and renal involvement is present in 90% and 80% of patients, respectively [10].

Despite the absence of formal diagnostic criteria for GPA, the American College of Rheumatology (ACR) has established classification criteria (see Table 2) which aid in distinguishing GPA as a separate entity from other forms of systemic vasculitis. A drawback of the criteria is that they do not differentiate GPA from microscopic polyangiitis or vasculitis mimics. Consequently, diagnosing GPA hinges on identifying a combination of clinical manifestations of systemic vasculitis, positive ANCA serology, and histological evidence of necrotizing vasculitis, necrotizing glomerulonephritis, or granulomatous inflammation from a relevant organ biopsy [1]. More recognized is the classic clinical triad which involves sinusitis, cough with hemoptysis, and glomerulonephritis [11]. Fortunately, the combination of histology from the skin biopsy highlighting the leukocytoclastic vasculitis and the positive serology for c-ANCA and PR3 and relevant pulmonary imaging aided in cinching the diagnosis.

Lung nodules and masses are the most common lesions, approximating 40–70% of patients [10]. Nodules are usually multiple and bilateral, with Guneyli et al. citing up to 17 nodules in a single individual [12]. Sizes range most commonly from 20 to 40 mm [13]. Cavitation is present in about 25% of lesions and poses a diagnostic quandary as they can be mistaken for metastases, lung abscesses, or septic infarcts [13]. Furthermore, pulmonary GPA lesions can also be spiculated, appearing similar to malignancy. Guneyli et al. have quoted spiculation in 22% of nodules or masses and 20% of patients, versus 6% and 17% by Lohrmann et al., respectively [12, 13].

Ground glass attenuation and consolidation are also common, occurring in up to 50% of patients with active disease. These usually develop as sequelae to alveolar hemorrhage [11]. Nevertheless, such radiology findings are more often diagnosed as common pulmonary syndromes such as pulmonary edema, acute respiratory distress syndrome (ARDS), bronchioloalveolar carcinoma, or pneumonia [10]. In fact, these were the differential diagnoses that were typical of our case, with a greater emphasis on ARDS developing as sequelae from a postobstructive pneumonia. As such, if these findings fail to ameliorate with standard therapy, an alternate diagnosis such as GPA should be entertained.

Postobstructive pneumonia is characterized by a proximal airway obstruction with ensuing infection of the distal lung parenchyma. The obstruction effectively retards the normal drainage mechanism of the lung, predisposing to a protracted course of infection, resistant bacteria, and extended courses of antibiotics [14]. Malignancy is the usual culprit, with traditional therapies being chemotherapy and external beam radiation therapy. Given that our patient was at a high operative risk, given her poor pulmonary reserve and deconditioning and ongoing heavy steroid use, concurrent chemoradiation was the modality employed in an attempt to obtain tumor control. However, these interventions often offer limited relief and predispose to multiple other obstacles, including cytopenias and endobronchial necrosis, inflammation, and swelling, which may convert a partial obstruction into a complete one [15]. As a result, this has inspired new developments in the field of interventional pulmonology, including endobronchial laser tumor treatment [16], electrocautery and argon plasma coagulation [17, 18], photodynamic therapy [19], cryotherapy [20], bronchoplasty [21], and airway stenting [22].

Distal airway involvement can also occur in GPA and is manifested by bronchial wall thickening and bronchiectasis [23]. Lobar or segmental atelectasis has been identified in one-fifth of patients [12]. Pleural nodules and pneumothoraxes are uncommon and if present should prompt investigation for concomitant infection or underlying malignancy [24]. Treatment of GPA ultimately results in fibrosis of the pulmonary lesions [25].

Without treatment, the mortality of GPA at 1 year was 80% [26]. Therapy has evolved over the years, from the introduction of glucocorticoids in 1948, to methotrexate, azathioprine, and cyclophosphamide in the 1960s and then plasma exchange in 1976 [6, 7]. Treatment strategies are focused on two major arms: remission induction and remission maintenance, with the aim of disease control while attempting to minimize drug toxicity.

The cyclophosphamide-glucocorticoid combination has usually formed the backbone for induction therapy. However, with the attendant toxicities associated with cyclophosphamide, there has been a shift to limit its exposure by switching to alternate immunosuppressants following remission after three to six months, using intravenous pulsed therapy versus oral continuous therapy, and even avoidance of cyclophosphamide altogether in early disease [27]. Adverse effects include immunosuppression and bone marrow suppression, opportunistic infections, hemorrhagic cystitis, bladder cancer, secondary malignancies, and infertility [28]. Mesna binds the toxic metabolite of cyclophosphamide and so lessens the risk for hemorrhagic cystitis [28].

Methotrexate is a reasonable substitute for less aggressive presentations in the absence of renal impairment [27]. It has been demonstrated to be as good as cyclophosphamide for remission induction and is given at a dose of 15–25 mg/week along with folic acid [29, 30]. That being said, methotrexate induction may be associated with a higher relapse rate as opposed to a cyclophosphamide-containing regimen [31].

Glucocorticoids are not employed as single-agent induction therapy; rather they are combined with other immunosuppressant therapies. Methylprednisolone 500–1000 mg intravenously daily for three days is followed by prednisolone 0.5–1 mg/kg/day for at least 4 weeks with a gradual taper to achieve the lowest dose effective at maintaining remission [1]. It is usually reduced to 10–20 mg/day by twelve weeks; however relapses are frequent during tapering [32, 33]. Unfortunately, the patient's final exacerbation coincided with the tapering of her steroid dose, as she was down to prednisone 7.5 mg daily. Furthermore, the flare was heralded by the onset of epistaxis a few days earlier which, in retrospect, should have prompted more aggressive therapy.

Rituximab (RTX), an anti-CD20 chimeric monoclonal antibody, provides an alternate option to cyclophosphamide in the induction therapy for GPA. It depletes circulating and tissue resident B cells by direct induction of apoptosis, complement-dependent cytotoxicity, and antibody-dependent cytotoxicity [34]. RTX is typically dosed at 375 mg/m^2^ once a week for four consecutive weeks [8, 9]. Based on the RITUXVAS and RAVE trials, RTX has shown being at least noninferior to cyclophosphamide in the treatment of newly diagnosed vasculitis. Notably, there were no differences in the time to remission for nephritis or pulmonary manifestations, and there were also no differences in the rate of severe adverse events between the RTX and cyclophosphamide groups [8, 9]. Mild-to-moderate infusion reactions can occur during or after treatment with RTX, and prophylactic intravenous methylprednisolone and antihistamine are recommended [35]. Hepatitis B (HBV) and hepatitis C (HCV) serologies should also be checked prior to initiating treatment as there is the possibility of reactivation of HBV and increased hepatic flares in HCV-positive patients [36, 37]. Other potential adverse effects include severe immunosuppression following B cell depletion with consequent risk for opportunistic infections, malignancy, and rarely progressive multifocal leukoencephalopathy associated with reactivation of the JC virus [1].

Plasma exchange (PLEX) deserves special mention in the induction phase of severe disease. The therapeutic mechanism may relate to expeditious elimination of ANCA from the peripheral circulation, with the caveat that there is also parallel depletion of clotting factors and circulating cytokines [1, 27]. PLEX may be prescribed for severe AAV alveolar hemorrhage alongside appropriate immunosuppressant agents, and it can also be used with cyclophosphamide for rapidly progressive, life-threatening renal vasculitis [38–40].

Despite effective induction therapy, 70% of patients will relapse and up to 20% will ultimately develop refractory disease [30]. As a result, maintenance therapy is commenced upon completion of the induction regimen and spans for a minimum of 12–18 months, with the goal of minimizing relapses and organ failure [1].

In an effort to spare cyclophosphamide exposure, sequential maintenance regimens that substitute cyclophosphamide with azathioprine or methotrexate have proven efficacious [32]. Rituximab has also demonstrated a reduction in relapse rate and prolongation of remission [41]. The role and timing of maintenance RTX become a provocative question in our patient's clinical course, given that her flare corresponded with the completion of the induction phase therapy.

Other agents that have been used in maintenance therapy include leflunomide and mycophenolate mofetil. Often, the immune suppressive agents are combined with low-dose corticosteroid. Withdrawal of the steroid component results in a higher relapse rate [30]. Additionally, persistent ANCA positivity following induction therapy is associated with an almost 80% relapse rate at 4 years compared with less than 20% for those who are ANCA negative [42]. For patients with a history of relapse, repeated courses of IVIg have permitted stable remission and reductions in immunosuppressive and glucocorticoids drugs [43].

With improved survival rates among patients diagnosed with GPA, there has been continuous evidence accrued hinting at an increased occurrence of cancer, including malignancies of the urinary bladder as well as the hematopoietic system, which has been attributed to cyclophosphamide [44]. A population-based cohort study conducted by Knight et al. investigated patients with GPA and the incidence of cancer among that population. The report revealed a markedly increased risk of cancer overall most pronounced for cancer of the bladder, nonmelanoma skin cancer (NMSC), leukemias, and malignant lymphomas [45]. Another study conducted by Fain et al. concluded that the cancers predominantly associated with the vasculitides were hematologic, including myelodysplastic syndrome and lymphoid malignancies, followed by solid tumors [46]. The study involved the analysis of sixty patients, of which 14 patients were diagnosed with vasculitis before the cancer, 24 patients were diagnosed simultaneously with both malignancy and a vasculitis, and 22 patients were diagnosed with a malignancy initially and a vasculitis thereafter. Faurschou et al. reported the results of a registry-based study on the long-term cancer risk following the conventional immunosuppressive therapy in GPA. They concluded that among those treated with a cumulative dose >36 grams of cyclophosphamide the overall cancer incidence increased, mainly due to an increased incidence of NMSC, bladder carcinoma, and myeloid leukemia [47]. However, there has also been a small reserve of cases highlighting the doubled or more risk for cancers in tissues involved in GPA, that is, the nose and middle ear, lung, and bladder. This occurrence may allude to the carcinogenic effect of the disease process itself [48, 49]. The disease burden as a risk for malignancy is difficult to separate from the effects of treatment in these patients, emphasizing the role for further research to elucidate the causes of cancer in this group.

A word on carcinoid tumors is as follows: Carcinoid tumors originate from enterochromaffin cells and are characterized by neuroendocrine differentiation. They are most commonly located in the gastrointestinal tract (68–74%) followed by the respiratory tract (25%) and very uncommonly within the thymus (2%) [50, 51]. Bronchial carcinoids constitute about 25% of all carcinoid tumors and about 2% of all primary pulmonary neoplasms [52]. Due to their highly varied morphologic and biochemical profiles, classification of the pulmonary neuroendocrine tumors has been established into four categories: low-grade typical carcinoid, intermediate-grade atypical carcinoid tumor, high-grade large neuroendocrine carcinoma, and small cell carcinoma [53]. Typical carcinoids are associated with a fairly benign behavior, versus atypical carcinoids which tend to be more aggressive and have a higher tendency to recur postoperatively. Atypical carcinoids also have a greater propensity for lymph node metastases, with rates quoted as high as 70% when compared to only 5% with typical carcinoids [54–56]. Atypical carcinoids demonstrate more modest 5-year survival rates (56–78%) as opposed to typical carcinoids which approach 100% [51, 57, 58]. Up to 92% of patients with pulmonary carcinoid tumors can be symptomatic, presenting with hemoptysis, cough, recurrent pneumonia, and unilateral wheezing [51]. Such clinical features overlap with those common to GPA and hence the diagnostic conundrum purported by that endobronchial carcinoid lesion. However, the incidence of a serotonin-provoked carcinoid syndrome is exceedingly uncommon (2%) in patients with lung or thymic carcinoids [57]. Complete surgical resection remains the gold standard for localized atypical carcinoid of the lung, with recommendations for adjuvant therapy for incomplete resection or more advanced pathologic stages [59]. Atypical carcinoid tumor above the diaphragm continues to be a rare entity, as such management continues to be dictated by small retrospective studies or serial case studies.

## 4. Conclusion

There are certain points that should be highlighted with this case. Firstly, though the adage follows that “common things are common,” this should not preclude one from exploring or, at least considering, alternate diagnoses. This becomes particularly evident when the proposed ailment does not improve with conventional therapy. As per our case, the patient was repeatedly treated for a postobstructive pneumonia, believed to be secondary to an obstructing endobronchial lesion. Furthermore, this marred her respiratory features, delaying the diagnosis of her underlying condition, that is, granulomatosis with polyangiitis. Secondly, the appropriate treatment at the appropriate time is paramount when coordinating care for complex disease processes. This patient received appropriate induction therapy, but in retrospect there could have probably been a greater emphasis on scheduling her maintenance therapy more expeditiously. Additionally, had the serology resulted earlier, there would have been less of a delay in initiating therapy and we may have been able to catch her in that critical window before she decompensated. Ultimately, this patient was concomitantly burdened with two severe conditions which made for a very intricate clinical course.

## Figures and Tables

**Table 1:** Organ-based clinical features of granulomatosis with polyangiitis.

Constitutional	Malaise, myalgia, arthralgia, anorexia, weight loss, and pyrexia
Mucocutaneous and orbital	Oral ulcers, oral granulomatous lesions, episcleritis, scleritis, conjunctivitis, keratitis, uveitis, retinal vasculitis, retinal artery or venous thrombosis, retinal exudates, retinal hemorrhages, blurred vision, blindness, proptosis, and orbital granulomatous masses

Cutaneous	Infarcts leading to ulcers and gangrene; leukocytoclastic vasculitis that may be found on biopsy

Ear and nose	Sensorineural and conductive hearing loss, persistent/recurrent nasal discharge +/− bloody, nasal ulceration, nose bridge collapse, nasal granulomatous lesions, and parasinus and sinus inflammation

Upper airway	Subglottic or tracheal stenosis, stridor

Lower airway	Cough, dyspnea, wheeze, hemoptysis, small airway obstruction, pulmonary infiltrates and hemorrhage leading to respiratory failure, and pleuritis

Renal	Hematuria, proteinuria, cellular cast on urine cytology; renal impairment in the form of acute kidney injury, chronic kidney disease, or end stage renal disease; diffuse pauci-immune crescentic necrotizing glomerulonephritis on biopsy

Cardiovascular	Occlusive vascular disease, pericarditis, pericardial effusions, cardiomyopathy, valvular heart disease, ischemic heart disease, and heart failure

Gastrointestinal	Peritonitis, bowel ischemia secondary to mesenteric vasculitis

Central and peripheral nervous systems	Headache, meningitis, seizures, cerebrovascular accidents, spinal cord lesions, cranial nerve palsies, sensory or motor peripheral neuropathy, mononeuritis multiplex, and cerebral mass lesion

**Table 2:** ACR classification criteria for granulomatosis with polyangiitis (formerly, Wegener's Granulomatosis).

Classification criteria	
(1) Nasal or oral inflammation	Painful or painless oral ulcers or purulent or bloody nasal discharge
(2) Abnormal chest radiograph	Pulmonary nodules, fixed pulmonary infiltrates, or pulmonary cavities
(3) Abnormal urinary sediment	Microscopic hematuria with or without red cell casts
(4) Granulomatous inflammation	Biopsy of an artery or perivascular area shows granulomatous inflammation

The presence of two or more of these four criteria yields a sensitivity of 88 percent.

The presence of two or more of these four criteria yields a specificity of 92 percent.
